# Facile Synthesis of Methylammonium Lead Iodide Perovskite with Controllable Morphologies with Enhanced Luminescence Performance

**DOI:** 10.3390/nano9121660

**Published:** 2019-11-21

**Authors:** Tao Wang, Huafang Zhang, Sumin Hou, Yan Zhang, Quanjun Li, Zhenlong Zhang, Huiping Gao, Yanli Mao

**Affiliations:** 1 School of Physics and Electronics, Henan University, Kaifeng 475004, China; wangtaoun@163.com (T.W.); hsm369688227@163.com (S.H.); zy2019un@163.com (Y.Z.); zhenlong2015@163.com (Z.Z.); gaohp@henu.edu.cn (H.G.); 2 Institute of Macro/Nano Photonic Materials and Application, Henan University, Kaifeng 475004, China; 3 State Key Laboratory of Superhard Materials, Jilin University, Changchun 130012, China; liquanjun@jlu.edu.cn

**Keywords:** organic–inorganic hybrid perovskites, synthesis, morphology control, MAPbI_3_, photoluminescence

## Abstract

Organic–inorganic hybrid perovskites with well-defined morphology have attracted much attention due to their unique photophysical properties. However, controlling the morphology of nanocrystalline perovskite to improve its photoelectric application remains a challenge. In this article, using a modified solution deposition method, we successfully synthesized uniform methylammonium lead iodide (MAPbI_3_) nanoplates, nanocubes, and nanorods and investigated the effect of morphology on the photoelectric properties of these materials. We found that the morphology can be controlled by regulating the amounts of reactant methylammonium iodide (MAI) and the rate at which MAPbI_3_ precursor is added into toluene solution, and that the corresponding size distributions can be optimized by tuning the final vacuum-drying temperature. The morphology has an obvious effect on the bandgap optimization and fluorescence enhancement of MAPbI_3_, and the nanoplates exhibit stronger photoluminescence intensity and have a longer carrier lifetime than nanocubes and nanorods. The results show that the morphologies of MAPbI_3_ perovskite nanocrystals can be controlled by tuning the synthesizing conditions, and the MAPbI_3_ perovskite nanocrystals with special morphology can be used in special nanosize optoelectronic devices.

## 1. Introduction

Hybrid organic–inorganic methylammonium lead halide perovskite, MAPbI_3_, has attracted extensive attention because of its excellent power conversion efficiency [1,2], high quantum yield [3], low-cost fabrication [4], and remarkable absorption property [5], and has been widely used in light-emitting diodes (LEDs) [6], lasers [7], and photodetectors [8]. In particular, recent work reports that the power conversion efficiency (PCE) of hybrid organic–inorganic perovskite solar cell has increased to nearly 23.3% [9], which is close to the PCE value (26.7%) of crystalline silicon solar cells [10]. MAPbI_3_ is expected to be the commercial material for the next generation of solar cells and optoelectronic devices due to these excellent properties [11]. However, most of the previous work has concentrated on MAPbI_3_ films, so there is a limited number of reports focusing on MAPbI_3_ nanocrystals, which present outstanding properties due to their larger spectral tunability and higher quantum efficiency [12]. Recent work shows that MAPbI_3_ quantum dots exhibit excellent tunable emission properties with high photoluminescence quantum yields of 46% [13] (higher than that of MAPbI_3_ films [14]), and good stability. MAPbBr_3_ nanoplates exhibit strong blueshift in the photoluminescence (PL) peak position, which is attributed to the quantum confinement in the strongly confined nanostructures [15]. Two-dimensional (2D) lead halide perovskite nanorods—(OA)_2_(MA)_2_Pb_3_(I*_x_*Br_1−*x*_)_10_—shift to higher energies in both absorbance and PL compared to the bulk material [16]. Moreover, the luminescent intensity and lifetime also changes in these different morphologies’ nanosamples [17], which has been attributed to the structural defects included in this perovskite [18]. These studies show that morphology plays an important role in tuning the absorption and PL of perovskite nanocrystals [19].

In recent years, researchers have attempted to prepare MAPbI_3_ nanocrystals with different morphologies, including zero-dimension (0D) nanodots, one-dimensional (1D) nanowires, nanorods, and nanobelts, 2D nanoplates, and three-dimensional (3D) nanocubes, by different preparation methods, including the dissolution–recrystallization method [20], conventional deposition method [21], and solution phase method [22]. However, the morphology of the prepared MAPbI_3_ nanocrystals are not uniform, with the coexistence of nanowires, nanorods, nanoplates, and nanobelts in the sample, which may reduce PL intensity and weaken light absorption, which will be detrimental to their application in nanodevices [23]. Recently, researchers further modified experimental approaches and obtained uniform MAPbI_3_ nanosamples. For example, Hintermayr et al. used ligand-assisted liquid-phase synthesized uniform MAPbI_3_ nanoplates with different thicknesses [24]. Wong et al., using the solution-phase anion exchange reaction method, prepared high-quality MAPbI_3_ nanorods arrays at room temperature [25]. Lan et al., applying the physical vapor deposition method, obtained freestanding layer-structured MAPbI_3_ nanoplates [26]. However, the synthesis process is complicated and slow, and it may be difficult to convert all of the initial reactants into the target products. Most recently, Fu et al. successfully synthesized single-crystal MAPbI_3_ nanorods and nanoplates via the dissolution–recrystallization method by tuning the precursor concentration of MAI, and found that the increase of MAI concentration causes the growth of perovskite nanorods and nanoplates with well-defined facets [16]. Zhu et al. has prepared the nanowires via the solution phase method, and regulated the aspect ratio of nanowires by adjusting the rate at which the MAPbI_3_ precursor solution is added to the toluene solvent [22]. Murugadoss et al. showed that the final annealing temperature also plays an important role in affecting the MAPbI_3_ morphologies in the anti-solvent assisted powder-engineering synthesized process [27]. Therefore, it can be inferred that the preparation conditions of MAPbI_3_ nanocrystals, such as the concentration of the precursor MAI in the preparation process, the rate of the precursor solution added to the disintegrating solvent, and the final annealing temperature, have important effects on the morphology. Simultaneously, we expect that the MAPbI_3_ nanocrystals with uniform morphology can be obtained by controlling these synthesizing parameters.

**Figure 1:**
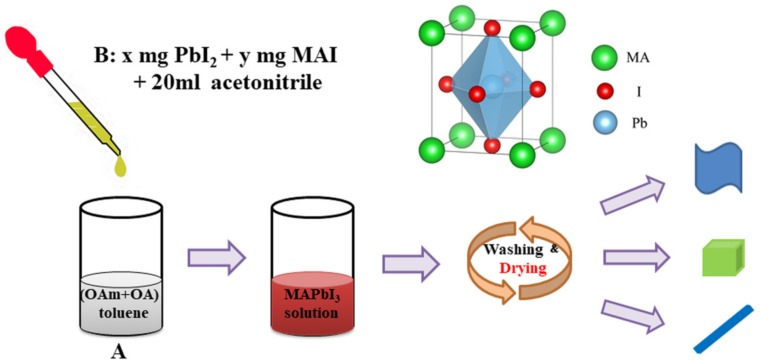
Schematic illustration for the formation of MAPbI_3_ perovskite nanoplates, nanocubes, and nanorods.

Herein, we used a modified solution deposition method to successfully synthesize nanoplates, nanocubes, and nanorods with uniform morphology MAPbI_3_ nanocrystals (shown in Figure 1) by regulating the concentration of MAI dissolving in acetonitrile, the rate at which MAPbI_3_ precursor is added into toluene solution, and the final vacuum drying temperature, and explored the effect of morphology on the photoelectric properties of materials. We find that concentration of MAI is an important factor in controlling the morphology of materials, and the drying temperature is a key cause of the size distribution of materials. By studying the photophysical properties of these three well-defined perovskite nanostructures, we find that the morphology has a great influence on the intensity of PL, carrier lifetime, and bandgap optimization of MAPbI_3_. These MAPbI_3_ nanocrystals with well-defined morphology show quite different fluorescent and optoelectric properties, which may expand their practical optoelectronic devices applications for further use.

## 2. Materials and Methods

### 2.1. Materials

Chemicals used include *N*,*N*-dimethylformamide (DMF, anhydrous, 99.8%, Aladdin Biotechnology Inc., Shanghai, China), oleic acid (OA, AR, Aladdin Biotechnology Inc., Shanghai, China), oleylamine (OAm, 80–90%, Aladdin Biotechnology Inc., Shanghai, China), lead iodide (PbI_2_, 99.99%, Xi’an Polymer Light Technology Corp, Xi’an, China), methylammonium iodide (MAI, 99.5%, Xi ’an Polymer Light Technology Corp, Xi’an, China), acetonitrile (GC, 99.5%, Aladdin Biotechnology Inc., Shanghai, China), and toluene (AR, 99.5%, Jiangsu, China) All chemicals were used as received without further purification.

### 2.2. Synthesis

MAPbI_3_ nanoplates, nanocubes, and nanorods were prepared via a simple modified solution deposition method. In the experiment procedure, 5.6 µL OAm and 10.4 µL OA were dissolved in 10 mL toluene as solution A, and *x* (*x* = 18.4, 36.8) mg lead iodide (PbI_2_) and *y* (*y* = 12.8, 18.4, 25.6) mg methylammonium iodide were dissolved in 20 mL acetonitrile as solution B. Then, 2.6 mL solution B was injected into solution A with different dropping rates under vigorous stirring. The solution turned red at the injection of solution A, indicating the formation of small-sized perovskite crystals. After stirring for 1 min, 15 mL toluene was added to the mixed solution dropwise, and kept stirring in the dark for 4 h. After the reaction finished, the obtained dark brownish suspension was recovered by centrifugation at 10,000 rpm for 10 min and washed three times with toluene, then dried in a vacuum-drying oven for 12 h.

### 2.3. Characterization

The powder X-ray diffraction (XRD) patterns were measured using a DX-2700 diffractometer (5.6 KW, 30 mA, Bruker Inc., Karlsruhe, Badensko-Wuertembersko, Germany) with a Cu Kα source (λ = 0.1542 nm) at 6.0 degrees/min and 2θ ranging from 10° to 50°. Ultraviolet and visible absorption (UV-Vis) spectra of the powder were acquired on a Varian Cary-5000 plus spectrophotometer (Agilent Inc., Sacramento, CA, USA). The steady-state photoluminescence spectra and the time-resolved photoluminescence measurements were carried out with a FLS980E spectrometer (Edinburgh Instruments Ltd., Scotland, UK), and a 515 nm diode laser with 1 W power was used to pump the samples. Scanning electron microscopy (SEM) was conducted using a field-emission scanning electron microscope (SEM, JEM-7001F, JEOL, Carl Zeiss Inc., Oberkochen, Baden-Württemberg, Germany) operated at 1.5 kV. High-resolution TEM (HTEM) images were measured using a transition electron microscopy instrument (TEM, JEM-2100, JEOL Ltd. Inc., Akishima, Tokyo, Japan) at an accelerating voltage of 200 kV.

## 3. Results

### 3.1. Influence of Reactant Concentration on Morphology of Resulted Samples

**Figure 2:**
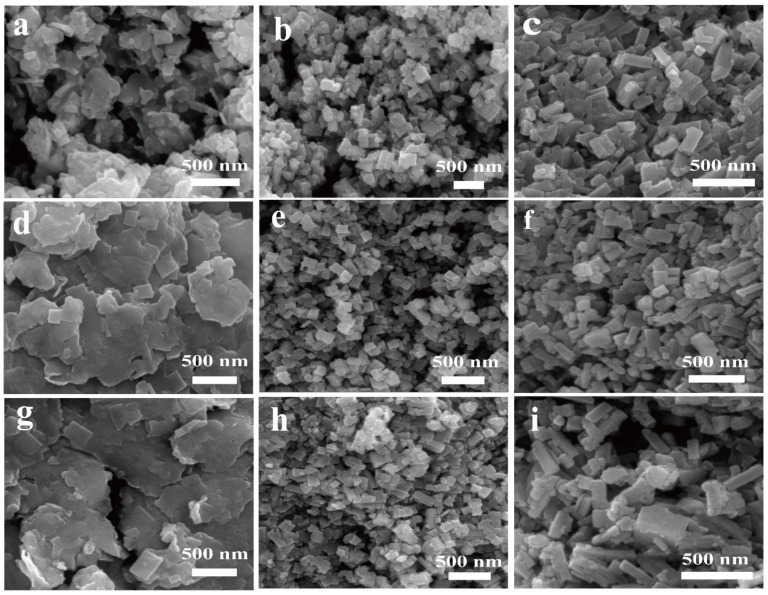
SEM images of the samples. Nanoplates, solution B: 18.4 mg PbI_2_, 12.8 mg MAI, 20 mL acetonitrile, dropping rate: 1 mL/min, dried at (**a**) 50 °C (Np-50), (**d)** 70 °C (Np-70), and (**g**) 95 °C (Np-95). Nanocubes, solution B: 18.4 mg PbI_2_, 18.4 mg MAI, 20 mL acetonitrile, dropping rate: 26 mL/min, dried at (**b**) 50 °C (Nc-50), (**e**) 70 °C (Nc-70), and (**h**) 95 °C(Nc-95). Nanorods, solution B: 36.8 mg PbI2, 25.6 mg MAI, 20 mL acetonitrile, dropping rate: 26 mL/min, dried at (**c**) 50 °C (Nr-50), (**f**) 70 °C (Nr-70), and (**i**) 95 °C (Nr-95).

By controlling the concentration of the MAI precursor in solution B, and the dropping rate of solution B into the solution A, MAPbI_3_ nanoplates, nanocubes, and nanorods perovskite were successfully synthesized. The SEM images of the as-synthesized perovskite nanocrystals are shown in Figure 2.

Figure 2a–c show the SEM images of the obtained sample synthesized with different amounts of MAI precursor. When solution B contained 18.4 mg PbI_2_ and 12.8 mg MAI, and the dropping rate of solution B into A was 1 mL/min, nanoplates were synthesized (Figure 2a). When the amount of MAI increased to 18.4 mg and the dropping rate of solution B into A was 26 mL/min, nanocubes were synthesized (Figure 2b). When the amount of MAI was further increased to 25.6 mg, and the dropping rate of solution B into A kept to 26 mL/min, nanorods were obtained (Figure 2c). These results suggest that the MAPbI_3_ nanoplates, nanocubes, and nanorods can be obtained by changing the amount of MAI in the synthesis process.

Yang and Fu et al. have reported that the crystallization of MAPbI_3_ is an in situ transformation process at low MAI concentration, where CH_3_NH_3_^+^ and I^−^ are directly embedded in the lead frameworks to form MAPbI_3_, and the resulted MAPbI_3_ samples could present the same original shape as PbI_2_ [28]. Meanwhile, it follows a dissolution−crystallization process at high MAI concentration. Iodide ions, acting as both lead ligands and reaction products at high MAI concentrations, can increase the solubility of PbI_2_ [28]. The presence of a large amount of PbI_4_^2−^ complex ions in the solution promotes the bonding between each complex and better coordinates the growth of the crystal. The following chemical equation is from [29]:

PbI_2_+ 2I^−^ ⇌ PbI_4_^2−^ (1)

CH_3_NH_3_^+^ + PbI_4_^2−^ ⇌ CH_3_NH_3_PbI_3_ + I^−^. (2)

In our case, we used PbI_2_ nanoplates (Inset of Figure S1a) for the synthesis of MAPbI_3_ samples, and thus obtained MAPbI_3_ nanoplates at a relatively low MAI concentration (12.8 mg). When the amount of MAI was increased to 18.4 mg, the growth of MAPbI_3_ followed a dissolution−crystallization process, and thereby promoted the formation of nanocubes. When the amount of MAI was increased to 25.6 mg, it is most likely that the crystallization of MAPbI_3_ continued along the growth direction of the nanocubes, and grew into nanorods.

### 3.2. Influences of the Dropping Rate on the Morphology of MAPbI_3_ Nanosamples

Figure S1 shows the SEM images for nanoplates, nanocubes, and nanorods synthesized with slow and fast dropping rates (1 mL/min and 26 mL/min). We noticed that the nanoplates were more uniform under slow dropping rates, whereas the nanocubes and nanorods were more uniform under fast dropping rates. As illustrated above, the crystallization of MAPbI_3_ nanoplates is an in situ transformation process, which is a rapid reaction process [28]. In the case of fast addition, the reactants could have already nucleated and grown before being diffused evenly in the solution. Therefore, parts of the nanoplates could grow into large pieces, whereas others could not, which results in non-uniform nanoplates with poor dispersion (Figure S1a). In the slow-addition samples, the concentration of the precursor was more uniform, leading to a more uniform nucleation, so well-dispersed nanoplates were obtained (Figure S1b). For the nanocubes and nanorods, the crystallization of MAPbI_3_ is a dissolution–crystallization process; therefore, the nucleation process is relatively sluggish [28]. Therefore, in the case of rapid addition, it is most likely that the reactants have diffused evenly in the solution before nucleating, and resulted in uniform nanocubes and nanorods (Figure S1c,e). In the slow-addition samples, the dropped reactants may continue to grow on the surface of the already-nucleated nanocrystals or form a new nucleation, so small particles will appear and result in non-uniform MAPbI_3_ samples. (Figure S1d,f).

### 3.3. Influence of Drying Temperature on the Size of Resulted Samples

In order to obtain more uniform materials, we studied the effect of drying temperature on the morphology of the final products. As shown in Figure 2a,d,g, the obtained nanoplates were dried at 50 °C, 70 °C, and 95 °C, respectively. After being dried at 70 °C, the surface of the nanoplates became smoother, and when the drying temperature rose to 95 °C, small nanoplates appeared on the surface of the sample. For the nanocubes (Figure 2b,e,h), we noticed that small nanoparticles appeared after drying at 50 °C. After drying at 70 °C, these small particles disappeared and the morphology of the material was more uniform. However, when the drying temperature rose to 95 °C, some of the nanocubes were transformed into nanorods, and small particles appeared in the sample again. For nanorods (Figure 2c,f,i), samples were uniform after drying at 50 °C. When the drying temperature increased to 70 °C and 95 °C, a large number of small particles appeared in the sample, and the uniformity of the sample was significantly worse.

**Figure 3:**
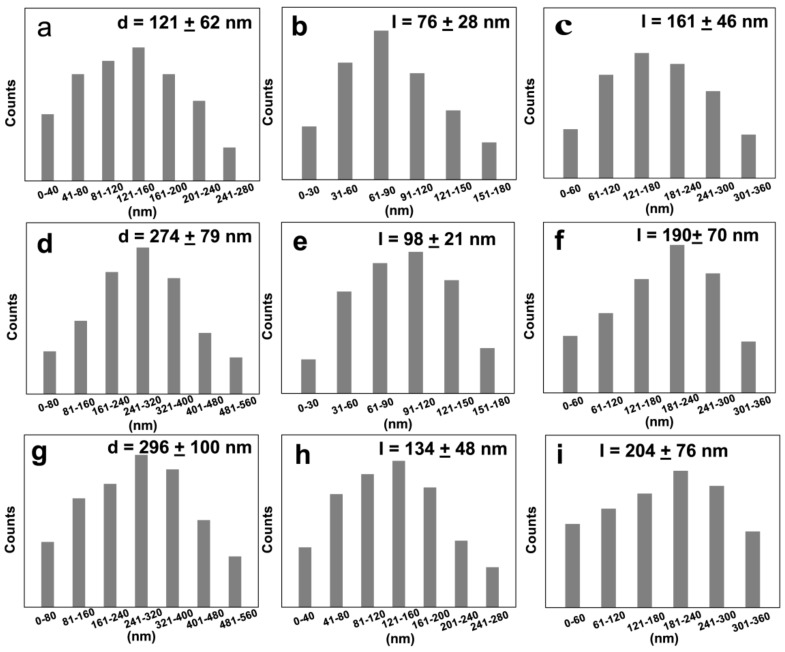
The corresponding size distribution of MAPbI_3_ nanoplates, nanocubes, and nanorods dried at 50 °C (**a**–**c**), 70 °C (**d**–**f**), and 95 °C (**g**–**i**).

To clarify the change of sample size with temperature, we made the size-distribution diagram shown in Figure 3. For the MAPbI_3_ nanoplates (Figure 3a,d,g), the average sizes were 121 ± 62 nm, 274 ± 79 nm, and 296 ± 100 nm after being dried at 50 °C, 70 °C, and 95 °C, respectively. For the MAPbI_3_ nanocubes (Figure 3b,e,h), the average sizes were 76 ± 28 nm, 98 ± 21 nm, and 134 ± 48 nm after being dried at 50 °C, 70 °C, and 95 °C, respectively. For the nanorods (Figure 3c,f,i), the average sizes were 161 ± 46 nm, 190 ± 70 nm, and 204 ± 76 nm after being dried at 50 °C, 70 °C, and 95 °C, respectively. We have resynthesized these samples more than three times, with the SEM images and the corresponding size distribution presenting high reproducibility (Figure S2). These results indicate that the size of the obtained samples increased as the drying temperature was increased, and MAPbI_3_ nanoplates, nanocubes and nanorods with a more uniform morphology were obtained after being dried at 70 °C, 70 °C, and 50 °C, respectively. According to early reports, the changes of size caused by changing the drying temperature may result from the excess surface energy gained from the lattice [30]. Therefore, we provide a facile and effective way to selectively synthesize uniform MAPbI_3_ nanoplates, nanocubes, and nanorods by controlling the drying temperature.

### 3.4. Analysis of the Crystal Structure

**Figure 4:**
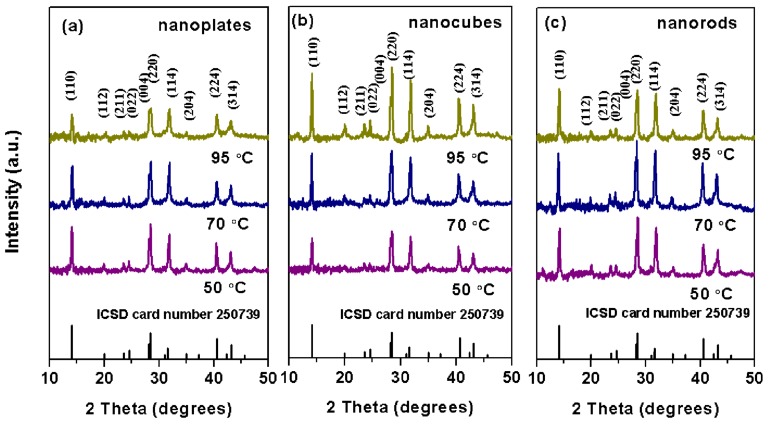
X-ray diffraction patterns of MAPbI_3_ (**a**) nanoplates, (**b**) nanocubes, and (**c**) nanorods dried at 50 °C, 70 °C, and 95 °C.

**Figure 5:**
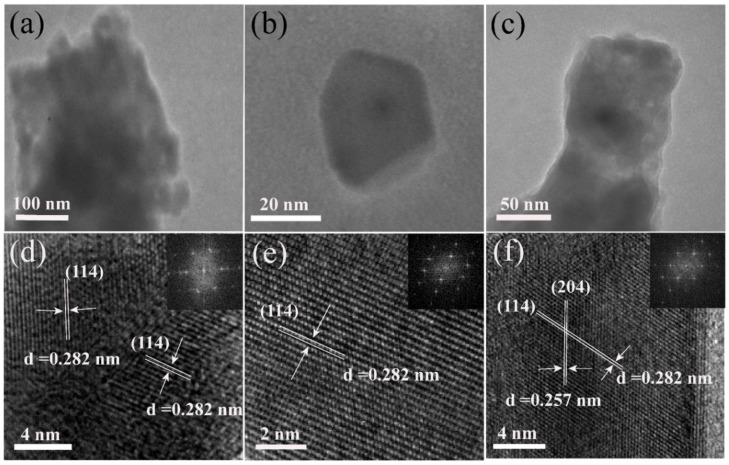
TEM image of MAPbI_3_ (**a**) nanoplates, (**b**) nanocubes, and (**c**) nanorods, respectively, and their high-resolution TEM (HRTEM) images (**d**–**f**). The inset is the corresponding fast Fourier transformation (FFT) pattern.

The XRD patterns of MAPbI_3_ nanoplates, nanocubes, and nanorods dried at different temperatures are shown in Figure 4. All the diffraction patterns match well with the standard pattern of MAPbI_3_ perovskite in the tetragonal phase with a space group of I4/mcm (ICSD card No.250739), suggesting that the drying temperature did not affect the lattice structure of the material. In addition, we noted that the nanoplates and nanocubes dried at 70 °C, and the nanorods dried at 50 °C, have stronger diffraction peaks, indicating that these materials have higher crystallinity. Figure 5 shows the TEM, the high resolution TEM (HRTEM), and the fast Fourier transformation (FFT) images of the MAPbI_3_ nanocrystals. In the HRTEM image of a MAPbI_3_ nanoplate (Figure 5d) and the FFT image (inset of Figure 5d), the interplanar distances of 0.282 nm, which corresponded to the (114) crystal faces of the tetragonal MAPbI_3_ structure, were clearly observed. These results may indicate that the (114) crystal plane growth direction was forbidden during the growth process of the nanoplates. In nanocubes (Figure 5e), the (114) crystal plane with an interplanar distance of 0.282 nm was also observed. However, in nanorods (Figure 5f), two interplanar distances of 0.257 nm and 0.282 nm were identified, which corresponded well with the (204) and the (114) crystal faces of the tetragonal MAPbI_3_ structure. As the (204) crystal face is parallel to the nanorods growth direction, and the [010] direction was perpendicular to the (204) crystal faces, it can thus be deduced that the nanorods may grow along the [010] direction.

### 3.5. Influence Morphology on the Optical Properties of MAPbI_3_ Nanosamples

**Figure 6:**
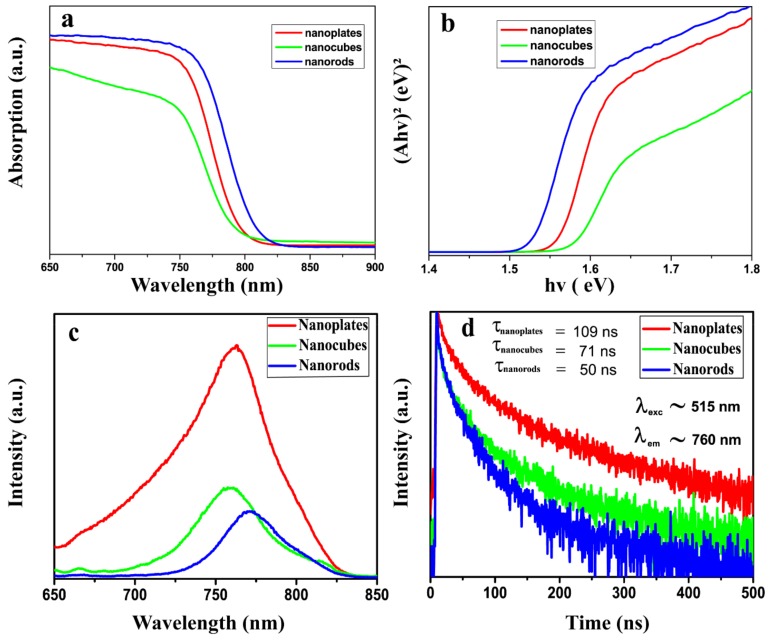
(**a**) Visible absorption spectrum, (**b**) direct bandgap tauc plots, (**c**) steady-state photoluminescence, and (**d**) time-resolved photoluminescence of MAPbI_3_ nanoplates, nanocubes, and nanorods.

In order to explore the effect of morphology on the bandgap and luminescence of MAPbI_3_, we further measured the absorption (Figure 6a), PL (Figure 6c), and PL lifetime (Figure 6d) spectra of the uniform MAPbI_3_ nanoplates, nanocubes, and nanorods, respectively. In Figure 6a, a slight shift of the absorption edges for the three different morphologies of perovskite was observed. Compared with MAPbI_3_ nanocubes, the absorption edge of MAPbI_3_ nanoplates underwent a slight red shift (about 8 nm), and the absorption edge of MAPbI_3_ nanorods presented a more significant red shift (about 22 nm). According to previous reports, the luminescence of MAPbI_3_ perovskite comes from the direct bandgap [31]. Therefore, we used a direct bandgap formula (Ahv)^2^ versus hv (where A = absorption coefficient, hv = energy of light), with the direct bandgap tauc plots obtained from absorption data (shown in Figure 6b). The calculated bandgaps for nanorods, nanocubes, and nanoplates were 1.53 eV, 1.58 eV, and 1.56 eV, respectively. MAPbI_3_ nanorods had smaller bandgaps than nanocubes and nanoplates, and had a larger absorption range under the illumination of sunlight, which was more conducive to the capture of sunlight. Figure 6c shows the PL spectra measured using a 515 nm laser source. The PL spectrum of MAPbI_3_ nanocubes, nanoplates, and nanorods shows a PL emission peak at about 759 nm, 763 nm, and 771 nm, respectively. The luminescence peaks were also red-shifted, which was consistent with the results of the absorption bandgap measured in Figure 6a. Therefore, we further performed PL lifetime measurements on MAPbI_3_ nanostructures (Figure 6d). The decay of the luminescence intensity was fitted with an biexponential equation [32,33], I(t) = I_0_ + A_1_ exp(−t/τ_1_) + A_2_exp(−t/τ_2_), where τ_1_ and τ_2_ are the short and long lifetimes, respectively, and A_1_ and A_2_ are decay amplitudes of the component. The obtained average lifetimes for nanocubes, nanoplates, and nanorods are 71 ns, 109 ns, and 50 ns, respectively, which were calculated based on the following equation τ = (A_1_ × τ_1_^2^+A_2_ × τ_2_^2^)/(A_1_ × τ_1_+A_2_ × τ_2_). We noticed that the PL intensity increased in turn in nanorods, nanocubes, and nanoplates, and the corresponding PL lifetime increased in turn for nanorods (50 ns), nanocubes (71 ns), and nanoplates (109 ns). These results indicate that nanoplates have stronger PL intensity and longer carrier lifetimes. The longer average lifetimes of MAPbI_3_ nanoplates indicate that the nonradiative loss of the excitons was reduced, which enhances the radiative recombination. As a result, the PL intensity is increased [34,35]. According to early reports, the optical properties of perovskites mainly depend on structural defects, such as Pb, I, and CH_3_NH_3_ vacancies [19]. The more structural defects in the material, the more excited electrons will transfer energy to the defects and return to the ground state by nonradiative transition, thus reducing the luminous intensity [36]. The preparation process has been suggested to be the key factor in determining the defect density in perovskites [16]. In our case, the crystallization process of nanoplates is a simple in situ transition process, and fewer defects could be preserved in the resulted nanoplates. Thus, the nanoplates present intense luminescence and long lifetimes. For nanocubes, more reactants were used in the synthesis process, and as the formation of nanocubes is a dissolution−crystallization process, it is most likely that parts of the unreacted reactants form vacancies in the resulting nanocubes, which decreased the luminescence and lifetimes. For nanorods, the amount of reactant was further increased in the synthesis process, resulting in more defects in the samples, and further decreasing the luminescence and lifetime of nanorods. The enhanced luminescence and lifetimes of nanoplates could result in higher carrier transport and longer electron-hole recombination lifetimes [24,32,34], thus exhibiting superior photophysical properties and semiconducting quality.

## 4. Conclusions

In conclusion, using a modified solution deposition method, we successfully synthesized uniform tetragonal MAPbI_3_ perovskite nanoplates, nanocubes, and nanorods by controlling the concentration of MAI precursor in solution B, the rate of B solution addition into A solution, and drying temperature. The calculated bandgaps for nanorods, nanocubes, and nanoplates were 1.53 eV, 1.58 eV, and 1.56 eV, respectively. Nanoplates exhibited stronger PL intensity and longer carrier lifetimes than nanocubes and nanorods. Further analysis indicates that the morphology-dependent physical properties could mainly relate to the defect content in these different-morphology MAPbI_3_ nanomaterials. These results suggest that the morphology plays an important role in tuning the bandgap, lifetime, and fluorescence intensity of MAPbI_3_. These results provide a facile method for controlling the morphologies of MAPbI_3_ nanomaterials, and a new way to tune the photoelectric properties of the material. This study thus opens new experimental quests on the basic electron transport and optical properties of different morphologies of MAPbI_3_ nanocrystals for expanding their practical applications in nanoscale optoelectronic devices.

## Supplementary Materials

The following are available online at https://www.mdpi.com/2079-4991/9/12/1660/s1, Figure S1: SEM images of the obtained samples. (a) Np-70, fast addition (26 mL/min) (b) Np-70, slow addition (1 mL/min) (c) Nc-70, fast addition (26 mL/min) (d) Nc-70, slow addition (1 mL/min) (e) Nr-50, fast addition (26 mL/min) (f) Nr-50, slow addition (1 mL/min). Inset of (a): SEM images of PbI_2_. Figure S2: SEM images of the obtained samples and the corresponding size distribution. (a) Np-70, dropping rate: 1 mL/min (b) Nc-70, dropping rate: 26 mL/min, (c) Nr-50, dropping rate: 26 mL/min.
