# Supplementary material for: Facile Synthesis of Methylammonium Lead Iodide Perovskite with Controllable Morphologies with Enhanced Luminescence Performance
Source: Nanomaterials (Basel). 2019 Nov 21;9(12):1660. doi: 10.3390/nano9121660 (PMC6956029; doi:10.3390/nano9121660)
Supplement: Supplementary file 1 [file nanomaterials-09-01660-s001.pdf]

Supplementary materials:

Facile Synthesis of Methylammonium Lead Iodide Perovskite with Controllable Morphologies with Enhanced Luminescence Performance

Tao Wang [1], Huafang Zhang [1,2,*], Sumin Hou [1], Yan Zhang [1], Quanjun Li [3], Zhenlong Zhang [1,2], Huiping Gao [1,2], and Yanli Mao [1,2,*]

[1] School of Physics and Electronics, Henan University, Kaifeng 475004, China; wangtaoun@163.com (T.W.); hsm369688227@163.com (S.H.); zy2019un@163.com (Y.Z.); zhenlong2015@163.com (Z.Z.); gaohp@henu.edu.cn (H.G.)

[2] Institute of Macro/Nano Photonic Materials and Application, Henan University, Kaifeng 475004, China

[3] State Key Laboratory of Superhard Materials, Jilin University, Changchun130012, China; liquanjun@jlu.edu.cn

* Correspondence: zhf610@163.com (H.Z.); ylmao@henu.edu.cn (Y.M.)

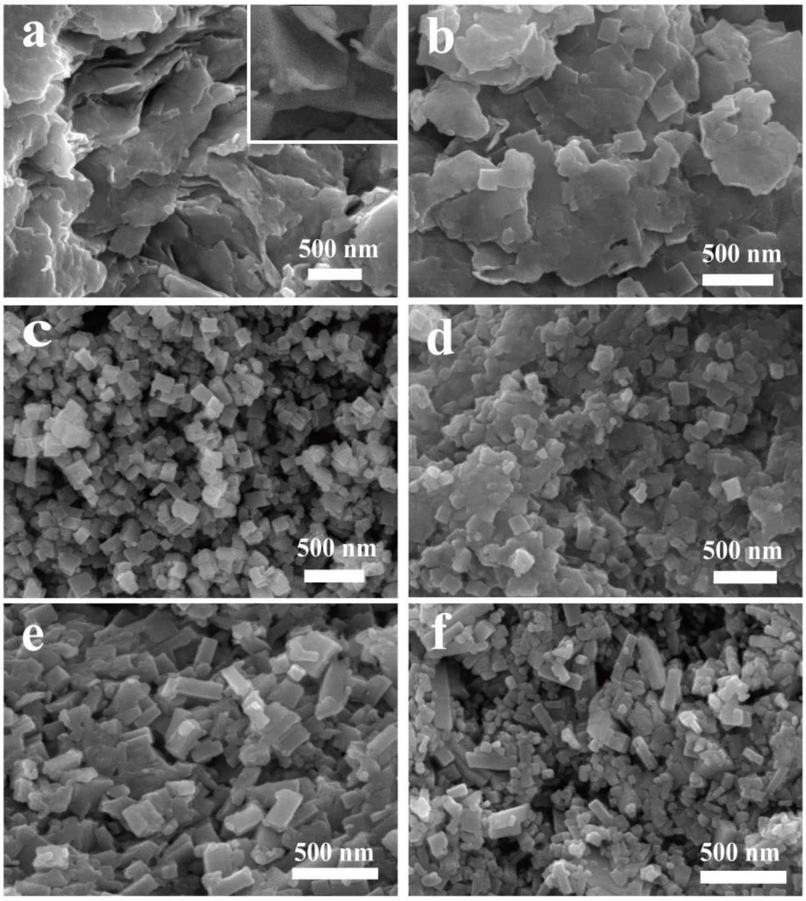

Figure S1. SEM images of the obtained samples. (**a**) Np-70, fast addition (26ml/min) (**b**) Np-70, slow addition (1ml/min) (**c**) Nc-70, fast addition (26ml/min) (**d**) Nc-70, slow addition (1ml/min) (**e**) Nr-50, fast addition (26ml/min) (**f**) Nr-50, slow addition (1ml/min). Inset of (a): SEM images of PbI_2.

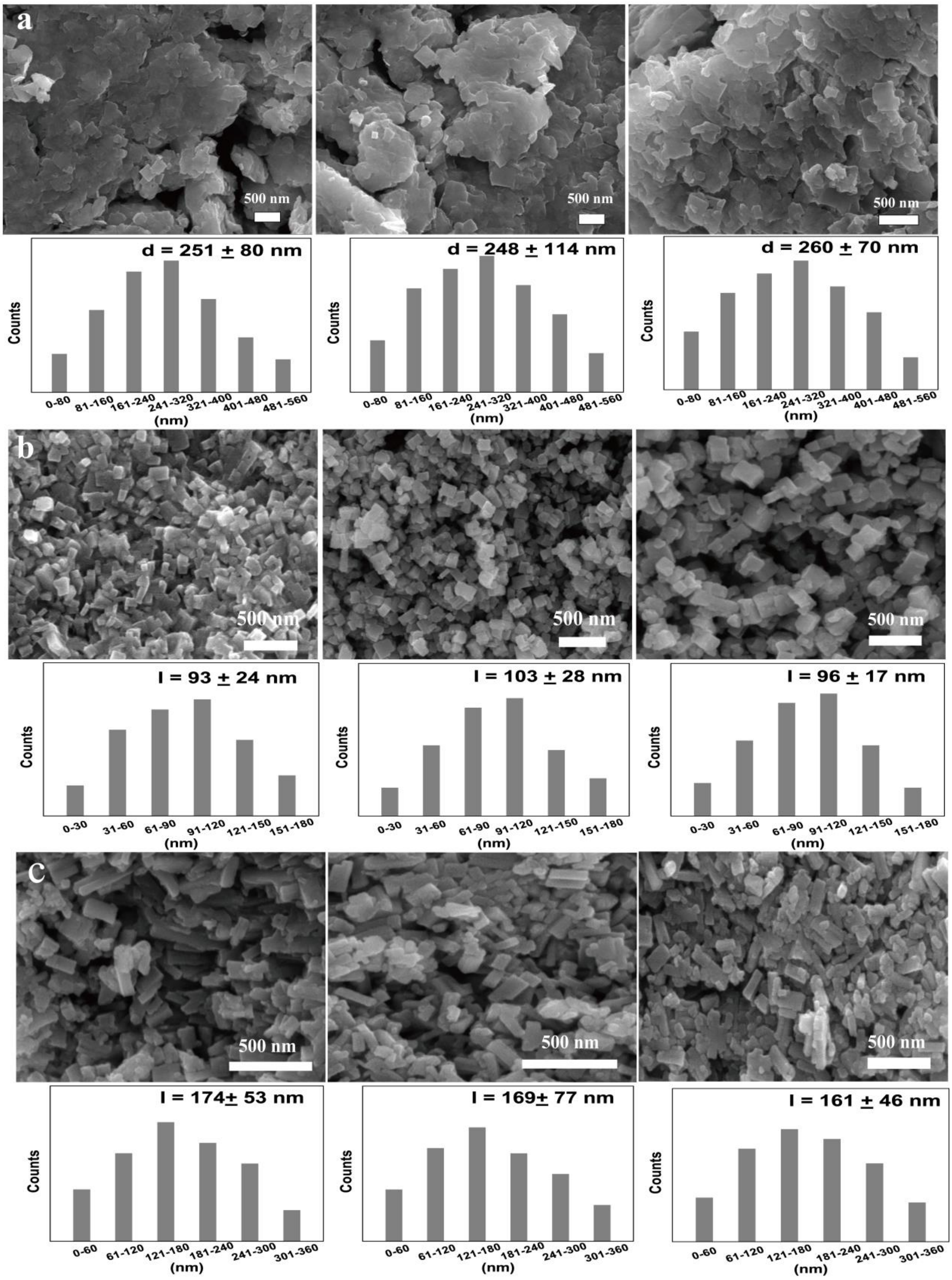

Figure S2. SEM images of the obtained samples and the corresponding size distribution. (**a**) Np-70, dropping rate: 1 mL/min (**b**) Nc-70, dropping rate: 26 mL/min, (**c**) Nr-50, dropping rate: 26 mL/min.

From Figure S2 can note that shapes of nanorods, nanoplates and nanocubes are similar, and the deviation of the measured size are less than 9%, 4%, 3%, respectively. And the size distributions are also stable.
